# Developmental Dyscalculia in Relation to Individual Differences in Mathematical Abilities

**DOI:** 10.3390/children11060623

**Published:** 2024-05-23

**Authors:** Ann Dowker

**Affiliations:** Department of Experimental Psychology, Oxford University, Oxford OX2 6GG, UK; ann.dowker@psy.ox.ac.uk

**Keywords:** developmental dyscalculia, individual differences, components of arithmetic, individual profiles, brain characteristics, domain specific, domain general

## Abstract

There is still much debate about the exact nature and frequency of developmental dyscalculia, and about how it should be defined. This article examines several key questions in turn: Is developmental dyscalculia a distinct disorder, or should it be seen as the lower end of a continuum—or possibly more than one continuum—of numerical ability? Do individuals with developmental dyscalculia show atypical brain structure or function? Does the study of acquired dyscalculia have anything to teach us about developmental dyscalculia? In studying dyscalculia, should we look less at arithmetical ability as a single entity, and more at separable components of arithmetical ability? How heterogeneous is developmental dyscalculia, and how important is it to study individual profiles? To what extent is developmental dyscalculia influenced by domain-specific versus domain-general abilities? The conclusion is that, though a significant amount has been discovered through existing research, and though this has some important implications for screening and diagnosis of dyscalculia, there is much more research that still needs to be conducted if we are to answer all of these questions fully. In particular, the study of developmental dyscalculia must be more integrated with the study of individual differences in mathematics in the population as a whole.

## 1. Introduction

Developmental dyscalculia is usually defined as significant difficulties in learning mathematics that are not the result of low IQ, sensory impairments, or lack of educational opportunity. There is still much debate about the exact nature and frequency of developmental dyscalculia. There are several important questions still to be answered, such as the following:

(1) Is developmental dyscalculia a distinct disorder, or is it better seen as involving the lower end of a continuum of numerical ability? If it is a distinct disorder, what are the characteristics that define it? Could it indeed be more than one distinct disorder? If it represents the lower end of a continuum, where should we place the cut-off point?

(2) Do individuals with dyscalculia show atypical brain structure or function?

(3) What characteristics, if any, does developmental dyscalculia share with acquired dyscalculia?

(4) Could there be several continua, involving different numerical abilities, given findings that suggest that arithmetical ability is componential and that “there is no such thing as arithmetical ability, only arithmetical abilities” [1]?

(5) How heterogeneous is developmental dyscalculia, and how important is it to study individual profiles?

(6) What domain-specific weaknesses are particularly relevant to developmental dyscalculia?

(7) How might weaknesses in domain-general abilities relate to developmental dyscalculia?

Such questions need to be considered in the wider context of the nature and extent of individual differences in mathematical difficulties.

### 1.1. Dyscalculia: Separate Condition or Lower End of the Continuum of Mathematical Attainment?

The difficulties in defining dyscalculia are in part embedded in the existence of a very wide range of individual differences in arithmetic in the general population. It is well known that people differ widely both in their level of arithmetical performance and in the nature of their relative strengths and weaknesses [1,2,3]. This leads to questions about whether dyscalculia is part of the range of individual variation in mathematical ability in the overall population or represents a separate condition.

Some of the ambiguities with regard to the deficits that underlie dyscalculia are the result of ambiguities about the criteria for diagnosing dyscalculia. Such ambiguities make it difficult to tell even how frequent dyscalculia is, let alone what its main foundations are. Studies have given widely different figures, ranging from less than 4% to over 10%, for the frequency of dyscalculia/mathematical learning disabilities [4,5,6,7,8,9,10,11]. The criteria used for defining dyscalculia, and for distinguishing it from the broader category of low attainment, usually fall into one or more of three categories [12,13,14]: (1) the specificity of mathematical difficulties, often based on the level of discrepancy between mathematical performance and IQ and/or other academic skills; (2) the severity of mathematical weaknesses; and (3) the persistence of mathematical weaknesses.

Most of the debates about the nature and frequency of dyscalculia, especially in relation to individual differences in the general population, relate either to specificity or severity. The issue of specificity will mainly be discussed later on with regard to the contribution of domain-general abilities. With regard to the severity criterion, it is difficult to distinguish dyscalculia from low attainment in arithmetic more broadly. Low attainment in arithmetic, often to a marked degree, is extremely common in many countries. Average levels of attainment in arithmetic vary significantly between countries [15], but low attainment can be observed in most countries. In the UK, nearly half of adults have only the level of mathematical knowledge of a primary school child [16]. About 20% have very severe numeracy difficulties, which make many everyday tasks difficult to complete successfully [17,18]. It would not usually seem appropriate to diagnose 20% or more or the population as having a specific learning disability. Distinguishing developmental dyscalculia from low attainment in mathematics is difficult even in high-income countries where most children attend school regularly. It is considerably more difficult in low- and middle-income countries [19].

Unsurprisingly, severity-based estimates of the frequency of dyscalculia vary according to the cut-off point used to determine low performance in mathematics, and both severity- and specificity-based estimates will vary according to the cut-off points used to determine high or low performance in IQ, reading, or other tests. For example, Dirks et al. found that about 10% of a sample of Dutch children met criteria for mathematical learning difficulties if these were diagnosed on the basis of their scoring below the 25th percentile on a mathematics test and above the 25th percentile on a test of language or reading [20]. Only 5.6% of the same children met the criteria when the 10th rather than the 25th percentiles were used. Some researchers have suggested that different criteria are appropriate for different purposes. Santos et al. have suggested that scoring 1.5 standard deviations or more below the mean on a mathematics test may be an appropriate criterion for educational and clinical purposes, while scoring below the 5th percentile might be a more appropriate criterion for research purposes [19].

### 1.2. Do Individuals with Dyscalculia Show Atypical Brain Structure or Function?

There is some evidence that individuals with developmental dyscalculia show differences from their typically developing counterparts in brain function and, to some degree, structure; however, most developmental dyscalculics do not have a history of brain damage, and there is no specific brain characteristic that is present in all individuals with developmental dyscalculia and none without developmental dyscalculia. It has to be remembered that there are significant individual differences within the population in brain characteristics, just as there are in cognitive characteristics.

Some studies suggest that, on average, the parietal lobes are smaller and less active in individuals with developmental dyscalculia. However, the differences in parietal activity could reflect, rather than causing, atypical or immature patterns of mathematical cognition. For example, Kucian, Grond, Rotzer, et al. found that children with developmental dyscalculia, like younger typically developing children, showed greater frontal and lower parietal activation than their typically developing age-mates [21]. However, when the children with dyscalculia were given an intervention that improved their mathematical performance, their brain activation patterns became more similar to those of their typically developing age-mates.

Some researchers suggest that people with dyscalculia differ from typically developing individuals not so much with regard to the structure or function of any given brain area, but with regard to the levels of connectivity between different brain areas. However, findings concerning the nature and even the direction of such differences are conflicting.

Kucian, Ashkenazi, Hänggi, et al. found that children with developmental dyscalculia showed reduced fibre projection between the frontal, parietal, and temporal regions and proposed that mathematical disabilities might be a function of reduced connectivity between different brain areas [22]. In contrast, Rosenberg-Lee, Ashkenazi, Chen, et al. carried out fMRI studies of children with dyscalculia and controls while carrying out addition and subtraction tasks, finding that those with dyscalculia showed excessive (rather than reduced) connectivity between the intraparietal sulcus and many other brain areas [23], Bulthé, Prinsen, Vanderauwera, et al. carried out functional MRI studies with 24 adults with a diagnosis of developmental dyscalculia and 24 matched controls [24]. When given tasks that involved non-symbolic magnitude representation, the individuals with dyscalculia showed less activation than the controls in the parietal, temporal, and frontal regions. They also showed hyper-connectivity in visual brain regions.

Thus, there is some evidence for differences in brain function between individuals with and without developmental dyscalculia, but some of the evidence is still quite conflicting. It is also often difficult to determine the direction of causation between brain differences and differences in mathematical performance.

### 1.3. Can Acquired Dyscalculia Tell Us Anything about Developmental Dyscalculia?

Developmental dyscalculia should not be equated with acquired dyscalculia, which is caused by brain damage, often to the parietal lobes. Developmental dyscalculia is very rarely caused by brain damage. Moreover, there is a fundamental difference between losing skills that one had previously acquired and experiencing difficulty in acquiring them in the first place. As Karmiloff-Smith pointed out, one cannot understand developmental disorders without viewing them in the context of the developmental process itself [25].

Nevertheless, there are some remarkable parallels among the findings about mathematical deficits in patients with acquired dyscalculia, findings about such deficits in individuals with developmental dyscalculia, and findings with regard to individual differences among typical individuals.

In acquired dyscalculia, there are very often marked discrepancies between different numerical abilities, and there is no single consistent hierarchy of abilities. Patients may be specifically impaired in a particular mode of representing numbers [26], and this is seen as important evidence for different modes of number representation being functionally independent with separable neural substrates. Dehaene and Cohen proposed the triple-code model, whereby numbers can be represented by Arabic digits, verbal number words, and analogue non-symbolic magnitude representations [27]. Patients may have specific impairments in any of these.

Patients are also often selectively impaired in specific arithmetical operations. For example, Van Harskamp and Cipolotti described three patients who were specifically impaired in basic addition, basic subtraction, and basic multiplication, respectively [28]. Venneri and Semenza (2011) reported a patient who showed deficits in multiplication but not in addition, subtraction or, interestingly, division [29]. Carota et al. reported a patient who could solve large-number subtraction without difficulty, provided that no borrowing was involved, but had severe difficulty with even small-number problems that involved borrowing [30].

Double dissociations have also been found in patients between exact calculation and estimation [31,32]. Patients also sometimes show dissociations among factual, procedural, and conceptual knowledge. For example, patients have sometimes shown preserved factual knowledge but impaired procedural knowledge, or vice versa [28,32]. Other patients may have impairments in both factual and procedural knowledge but not in conceptual knowledge, or in conceptual knowledge but not in factual or procedural knowledge [32,33,34,35].

Patients also show dissociations between subitising and other aspects of mathematics. Double dissociations have been found between subitising and counting [36,37]. Gosling, Demeyere, and Dowker (2023) studied 11 chronic patients with various sites of brain injury and found that there was no overall significant correlation between subitising speed and reaction time for simple addition or multiplication [38]. Two of the patients showed significant impairment in subitising, of whom one also showed impairment in addition but the other did not.

One of the largest-scale studies of mathematical abilities in patients with brain injury was carried out by Cappelletti, Butterworth, and Kopelman [39]. Thirty-six patients with either neurodegenerative disorders or focal brain lesions were compared with forty healthy controls on a set of numerical and calculation tests. Some but not all of the patients with focal lesions had damage to the parietal lobes. In fact, there was a less sharp distinction than expected between patients with and without parietal damage. All patients in this study, including those with parietal lesions, showed unimpaired processing of number quantity. Most patients with focal brain lesions, whether these involved the parietal lobes or not, showed impaired calculation skills, though most patients with neurodegenerative disorders showed unimpaired calculation skills.

It might be thought that patients would show impairments in and dissociations between specific components of arithmetic that would not be found in typical individuals or even in those with developmental dyscalculia. In fact, while patients do tend to perform worse in mathematics than healthy controls, dissociations can be found in apparently typical individuals if one looks for them. For example, Dowker [1,40] reported a healthy undergraduate student in a science subject, with an A grade in A-level mathematics, who generally performed very well in tests of calculation but showed a specific inability to carry out subtraction problems involving borrowing, somewhat similar to Carota et al.’s patient [30]. Dowker (2005) also reported a highly educated adult participant who had struggled with school mathematics but did have the equivalent of O-level mathematics [1]. She was given many of the same tests as Warrington’s patient [32] and showed a very similar pattern of relatively poor calculation but good arithmetical reasoning and definitions of arithmetical operations.

Thus, the study of patients with acquired dyscalculia provides strong evidence for the functional independence of different components of mathematical ability. Though dissociations between components are often particularly striking in such patients, similar dissociations can often be found in developmental dyscalculics, and even in typically achieving individuals. The study of such patients is thus relevant to our understanding of developmental dyscalculia, not because the physical causes are likely to be at all similar, but because of what they tell us about the componential nature of mathematical ability.

### 1.4. How Does Dyscalculia Relate to the Componential Nature of Arithmetical Cognition?

One key issue in determining the nature and frequency of dyscalculia is that arithmetical cognition is not a single entity but is composed of multiple components, according to Dowker [1,40,41]. Children most commonly have difficulties in only some of these components, rather than in all aspects of arithmetic [42,43,44], Gifford and Rockliffe [44] (p. 21) found that, in one group, “no pure cases [of dyscalculia] were found, although the children presented complex patterns of learning difficulties and compensatory strategies. The range of contributory factors suggests the need for new theoretical perspectives to consider learning difficulties and the need to study individual mathematics learning trajectories”.

The evidence is that numerical abilities are already componential in the preschool years. There have been a few studies of the structure of mathematical abilities in preschoolers. Purpura and Lonigan studied 393 preschool children and found that their informal numeracy skills could be divided into three factors: numbering, relations, and arithmetic operations [45]. The factor structure was the same in younger and older preschool children.

Dowker studied relationships and discrepancies between counting procedures and concepts in preschoolers [46]. She gave 80 four-year-old children several numerical tasks. These included (1) counting objects; (2) Wynn’s cardinal word principle task, involving counting versus grabbing [47]; (3) an order irrelevance task, where children counted a set of objects and were then asked how many they would get if they counted the objects in the other direction; (4) a task where they started with five objects and were then repeatedly asked to tell, without counting, how many objects there were after a new object was added; and (5) a task where they started with 10 objects and were then repeatedly asked to tell, without counting, how many objects there were after an object was removed. There were significant correlations between scores on most tasks, but individual children could show discrepancies in both directions between almost any two tasks. For example, proficiency in counting objects correlated significantly with success on the cardinal word principle task, but 22% of proficient counters failed the cardinal word principle task, and 41% of non-proficient counters passed the cardinal word principle task.

Studies of primary school children’s arithmetic also give strong evidence for the componential nature of mathematical abilities. Dowker [1,43,48] studied 291 unselected primary school children between the ages of 5 years 2 months and 9 years 10 months. She gave the children three different types of addition task: calculation, derived fact strategy use, and arithmetical estimation. The calculation task involved giving oral answers to problems of varying levels of difficulty, presented simultaneously in oral and written form. The difficulty of the arithmetic problems given to individual children in the estimation and derived fact strategy tasks was adapted to their assessed calculation performance levels in the calculation task.

In the derived fact strategy task, children were given the answer to an addition problem and then asked to solve another problem that could be solved quickly by using this answer, together with an arithmetical principle being investigated, e.g., commutativity, the t addition/subtraction inverse principle, and the fact that adding 1 to an addend will add 1 to the answer to the problem..

In the addition estimation task [43,49], each child was presented with a set of addition problems selected to be just too difficult for them to solve by mental calculation. Each set included nine addition sums to which a pair of imaginary characters (“Tom and Mary”) had estimated answers. The children were asked to evaluate each of the guesses on a five-point “smiley faces” scale ranging from “very good” to “very silly” and were themselves asked to suggest “good guesses” for each of the sums. Each set of “Tom and Mary’s” estimates included three good estimates (e.g., “7 + 2 = 10”, “71 + 18 = 90”), three estimates that were too small, and three estimates that were too large.

The addition calculation performance level correlated significantly with scores on both the estimation and derived fact strategy tests, despite the fact that the difficulty of the sums in the latter tasks was adapted to children’s pre-assessed addition calculation performance level. Even after controlling for calculation performance level, there was a strong independent relationship between derived fact strategy use and estimation. Nevertheless, there were individual children who showed marked discrepancies, in both directions, between scores on all possible pairs of arithmetic tasks.

Dowker [50] gave the same calculation, derived fact strategy, and estimation tasks to 204 children who had been identified by their schools as having arithmetical difficulties (not necessarily dyscalculia). These children were compared with 135 unselected children of similar age from the same schools. The unselected children performed very similarly to those in the previously mentioned studies. The children identified as having arithmetical difficulties performed less well on all tasks than the unselected children. They also showed even greater discrepancies between different tasks than did the unselected children. For example, the children with arithmetical difficulties showed no significant correlation between calculation, derived fact strategy use, and estimation. Moreover, scores on standardised tests of arithmetic—the WISC Arithmetic subtest and the British Ability Scales Basic Number Skills test—correlated significantly with both derived fact strategy use and estimation in the unselected group, but not in the group with arithmetical difficulties. The group differences were not due to ceiling or floor effects in either group, as both groups showed considerable variance in their scores on all tasks.

Thus, it appears that discrepancies between different components of arithmetic may be even greater in children with arithmetical difficulties than in typically achieving children. It may be the case that, though arithmetical cognition is componential in the population as a whole, the components are more integrated in typical arithmetical development than in children with arithmetical difficulties. Perhaps, in children with arithmetical difficulties, either weaknesses in individual components impede integration, or weaknesses in the integrative process impede progress in individual components, or both.

### 1.5. How Heterogeneous Are Deficits in Dyscalculia? The Importance of Studying Individual Profiles

There have been several studies that indicate that children with mathematical disabilities are not a homogeneous group and, if investigated for performance on numerous tasks, can be found to have very different profiles. Some of this heterogeneity is related to the level of severity of the mathematical difficulties, resulting in different patterns being found, depending on the cut-off point used for diagnosing mathematical disability [51]; some of it is due to the extent to which individuals’ mathematical disabilities are specific versus having comorbidities; and some of it is due to the componential nature of arithmetical ability.

One of the most extensive studies of profiles of children with mathematical difficulties was carried out by Bartelet et al. [52], who found six profiles: one of children with problems with non-symbolic number comparison, one of children with problems with mapping between symbolic and non-symbolic representation, one of children with counting difficulties, one of children with domain-general memory problems, one of children with a variety of domain-general cognitive deficits, and one of children with no obvious cognitive deficits. Salvador et al. found two main profiles: one of children with weak number sense and poor symbolic and non-symbolic comparison, and one of children with weak working memory: in other words, a “domain specific” and a “domain general” group [53]. Pieters et al. found one group with problems with arithmetical fact retrieval and one with problems with procedural calculation [54]. The first group tended also to have problems with motor skills, and the second with visual–motor integration. The latter deficits could be more a part of a comorbid disorder than of the mathematical disability itself.

Skagerlund and Träff studied children between the ages of 10 and 13 who were receiving special instruction in mathematics due to struggling with the subject [55]. They investigated two groups: One group of 16 children had particular difficulty with arithmetic fact retrieval and scored below the 5th percentile in a fact retrieval test but at or above the 15th percentile in tests of calculation. The other group of 34 children had a more general difficulty with arithmetic and scored below the 5th percentile in tests of both fact retrieval and calculation. Both groups were compared with 27 typically achieving pupils in the same classes. The children who had problems specifically with fact retrieval performed significantly worse than the typically achieving children on symbolic number naming and comparison tasks, but they performed similarly on non-symbolic number estimation, discrimination, and comparison tasks. The children who had problems with all arithmetic tasks performed significantly worse than the typically achieving children on both symbolic and non-symbolic number tasks.

Karagiannakis, Baccaglini-Frank, and Papadatos [56] proposed four main subtypes of mathematical disabilities: those involving deficits in core number skills, those involving deficits in memory retrieval and processing, those involving deficits in reasoning, and those involving deficits in visuospatial abilities. Only the first subtype might be seen as involving a specific mathematical disability, as opposed to being secondary to other deficits, but even the first type could be divided into several different deficit types, as classified by Andersson and Ostergren [57]: deficits in the ANS, deficits in the object tracking system (a mechanism which they considered to underlie subitising); deficits in numerosity coding (representation of quantities by symbols), deficits in access (comprehension of symbols and relating them to quantities), and any combination of the above.

Munez, Bull, Lee, and Ruiz studied 428 Singaporean children with low mathematical attainment and found two main groups: those who had specific difficulties with arithmetical fluency, and those who had more general difficulties with a broader range of arithmetical tasks [58]. Those with more general difficulties performed significantly worse than those with specific fluency difficulties on number line estimation, numerical discrimination, working memory, and reading. The groups did not differ in gender composition, socio-economic status, or age. Moreover, there were some differences between the groups as regards which factors predicted mathematics performance in a logistic regression. In the group with more specific fluency difficulties, the main independent predictors of mathematics performance were reading, working memory, and number line performance. In those with more general arithmetical difficulties, the main independent predictors of mathematics performance were reading and non-symbolic numerical discrimination.

Jordan, Hanich, and Kaplan [59] compared second-graders (7-to-8-year-olds) with difficulties in just mathematics, those with difficulties in both mathematics and reading, those with difficulties in just reading, and those who were achieving typically in both subjects. Both groups with mathematical difficulties performed worse in most areas of arithmetic. The children with only mathematical difficulties performed better in exact mental calculation and problem solving than the children with both mathematical and reading difficulties. The two groups with mathematical difficulties performed similarly on written calculation, place-value understanding, and approximate arithmetic.

Träff, Olsson, L Östergren, and Skagerlund, K. found considerable variability in profiles even when studying just four children with mathematical learning disabilities [60]. All had some working memory difficulties. Two appeared to have deficits involving the ANS: one mainly had symbolic deficits; and the other had no deficits in core numerical abilities, but only in working memory.

Huijsman, Kleemans, van der Ven, and Kroesbergen found it difficult even to subdivide children with mathematical difficulties into broad profiles, because they showed so much individual variation [61]. They studied 281 fourth-grade pupils in the Netherlands and tested them on a variety of mathematical skills (basic arithmetic, number sense, advanced mathematics) and a variety of domain-general abilities (non-verbal reasoning, working memory, phonological awareness, rapid naming). There was a “low achieving” group, who were the bottom third of the pupils in the mathematics tests, and who performed less well on average in both the mathematical and domain-general tests than did the higher achieving pupils, but did not show a consistent pattern of low performance on any specific test. Children who scored poorly both on mathematics and non-verbal reasoning did not differ significantly on any other domain-general cognitive test from children who scored poorly on mathematics but well on non-verbal reasoning.

Haberstroh and Schulte-Körne carried out a meta-analysis of 34 studies comparing the cognitive profiles of 8-to-12-year-old children with and without mathematical disabilities [62]. Mathematical disabilities were defined as mathematics test scores at or below the 16th percentile, without low IQ or other comorbid disorders. In all, the analysis included 680 children with mathematical disabilities and 1565 controls. The children with mathematical difficulties performed significantly worse than the controls in calculation, arithmetic fact retrieval, number sense (including quantity processing, quantity–number linking, and numerical relations), and visuospatial short-term storage. They did not show deficits in reading or related skills, though this may reflect the fact that the study specifically excluded children with comorbid reading disabilities.

Though the findings are quite complex and varied, they appear overall to support the conclusions of Fias, Menon, and Szucs [63], who argued that, on both a behavioural and a neural level, dyscalculia is best regarded as a heterogeneous disorder involving multiple components, rather than as a single deficit. They reviewed numerous fMRI studies that indicate that multi-componential neural networks subserve number processing and arithmetic. They considered that impairments in any of these components can lead to arithmetical deficits. This view appears to be consistent with the evidence from multiple sources.

### 1.6. Domain-Specific Factors in Typical Arithmetical Development and in Dyscalculia

Thus, a lot more research is needed about the causes and nature of dyscalculia. It is certainly true that some individuals have severe difficulties with numeracy and arithmetic, and they experience severe and lasting deficits in these areas in comparison with the majority of individuals. It is also true that, while educational and other environmental factors, emotional factors such as mathematics anxiety, and domain-general cognitive weaknesses have been found to contribute to mathematical difficulties, they cannot fully explain them. Mathematical difficulties must be partly explained by domain-specific factors.

Yet so far, no studies have consistently found a single precursor, or even correlate, of such mathematical difficulties, or indeed have reached a definitive conclusion as to what are the key domain-specific factors in numerical cognition as a whole. Influential models include Butterworth’s theory of the key importance of subitising [64] and Dehaene and Cohen’s [27] triple-code model of separate number representation systems by Arabic digits, verbal number words, and analogue non-symbolic magnitude representations (the latter often being referred to as the Approximate Number System or ANS).

Some studies, but not all, have found subitising to be a key deficit in individuals with mathematical difficulties. Some, but not all, have found approximate quantity estimation and comparison (corresponding to Dehaene and Cohen’s analogue magnitude representation system) to be a key deficit in individuals with mathematical difficulties. Some, but not all, have found symbolic number representation and arithmetic to be the chief or only deficit in individuals with mathematical difficulties.

The biggest debates are perhaps the following:

(1) Between those who consider that children with dyscalculia are impaired in foundational, non-symbolic quantity recognition and those who consider that their deficits are in symbolic number processing and, therefore, only appear when numeral recognition and processing start to develop in typically developing children.

(2) Within the group that emphasises foundational non-symbolic processing, between those who emphasise the exact recognition of small quantities (subitising) and those who emphasise the approximate recognition and comparison of larger quantities (the ANS).

Findings have been quite conflicting. Some studies have indicated that dyscalculic children are impaired in subitising [65,66,67,68,69,70]. One particularly large-scale study that reached this conclusion was carried out in Cuba by Reigosa-Crespo et al. [71]. This study involved 11,652 children in grades ranging from 2nd to 9th; 3.4% of these children showed deficits in basic numerical capacities, including subitising. Almost all of these children also had difficulties in calculation. An additional 9.35% had deficits in calculation, without the deficits in basic numerical abilities. Those who did have deficits in basic numerical abilities tended to have more severe calculation difficulties than those who had with poor calculation without the basic numerical deficits. Estevez-Perez et al. followed up this study [72] and found that subitising, verbal counting, and numerical magnitude comparison all predicted early and later acquisition of arithmetic and were impaired in children with low arithmetical attainment, including those with a diagnosis of developmental dyscalculia. The children with both low arithmetical achievement and poor subitising were slower at arithmetic and relied more on compensatory strategies than children with similarly low arithmetic scores who showed no subitising deficits.

Some other studies, however, have suggested that dyscalculic children are impaired in ANS measures but not in subitising [73,74,75,76,77], Anobile et al. [78], who looked at the entire range of arithmetical ability rather than dyscalculia specifically, found that simultaneous and sequential subitising did not correlate significantly with one another in primary school children [79,80,81]. Neither type of subitising predicted either children’s mental calculation or their digit magnitude knowledge. In contrast, estimation of larger numerosities did predict children’s arithmetic.

However, other studies have shown little or no impairment in non-symbolic number processing but significant impairment in symbolic number processing ([82]; also, see [83] for similar results in adults with a diagnosis of developmental dyscalculia).

Olsson, Ostergren, and Traff [84] studied 24 Swedish third-grade pupils with a diagnosis of developmental dyscalculia and compared them with controls matched for chronological age, gender, time of testing, non-verbal intelligence, and reading ability. Two controls were matched to each dyscalculic child, so that there were 48 controls in all. They were given tests of subitising small sets, enumerating larger sets, and non-symbolic and symbolic number comparisons. The dyscalculic children performed significantly less well than the controls on all of these tests, but they were particularly impaired on the symbolic tests.

The extent of the relationship between non-symbolic and symbolic quantitative skills is controversial. Views range from the idea that symbolic number processing and arithmetic arise directly from, and depend completely on, non-symbolic quantity processing, to the idea that they are largely independent processes. The results of most studies indicate that there is at least some relationship between them. Studies tend to suggest that this relationship diminishes over time, with symbolic and non-symbolic quantitative skills becoming increasingly mutually independent as children grow older [73,85,86,87]. This increasing mutual independence has been termed “symbolic estrangement” [88]. The explanation may be that symbolic abilities initially derive from non-symbolic abilities but then develop into a separate form of processing. Alternatively, or additionally, this “estrangement” may reflect changes with age in the nature of the symbolic abilities that are emphasised. Non-symbolic abilities may be more closely related to oral processing of number words than to the processing of written numerals.

It is also likely that, even if we consider only domain-specific abilities, there are multiple components of numerical ability that may be selectively impaired. One question that arises is why sometimes a seemingly selective deficit may be associated with globally poor performance in mathematics, while sometimes it does not appear to be associated with significant mathematical disability. Is this because some components are stronger prerequisites than others for arithmetical ability as a whole? Is it because of the contribution of domain-general abilities, and, if so, which? Is it because of environmental factors such as the level of support from family or school or, alternatively, the negative effects of being labelled as “bad at maths”, experiencing anxiety-provoking pressures and criticism from others, or exposure to negative attitudes to mathematics? Linked to the above, to what extent may an individual’s emotions and attitudes toward mathematics moderate the extent to which a specific weakness affects their mathematical performance as a whole?

### 1.7. Domain-General Factors in Typical and Atypical Arithmetical Development

One important issue, relevant both to our understanding of dyscalculia and of the broad spectrum of individual differences in mathematics, is the extent to which numeracy is related to domain-general versus domain-specific abilities in typical and atypical development. Domain-general abilities appear to play an important role, and to interact with domain-specific abilities, in an even more complex way with regard to components of arithmetical cognition in a broader sense. There are several domain-general abilities that have been found to be related to mathematical development. These include language, spatial abilities, attention, working memory, and executive functions such as inhibition.

Language has been found to be related, both cross-sectionally and longitudinally, to mathematical development and performance. Some mathematical skills seem to be more dependent on language than others. Multiplication appears to be more dependent on language than other arithmetical operations, probably because it is usually taught in a way that places greater emphasis on verbal memorisation. Guez, Piaz, Pinheiro-Chagas, et al. [89] found that preschool children’s language skills, but not their visuospatial skills, predicted their multiplication performance at the age of 11. In contrast, their visuospatial skills, but not their language skills, predicted their addition and subtraction performance at the age of 11. There is also evidence that different aspects of language differentially predict different aspects of mathematics. Phonological awareness is related to procedural aspects of arithmetical calculation [90,91], while semantic comprehension is more related to word problem solving [92,93,94].

There are consistently found to be associations between language disabilities and mathematical disabilities. Although dyslexia and dyscalculia are separate conditions and often occur independently, they have been found to co-occur considerably more often than would be expected by chance alone [6]. Indeed Moll, Landerl, Snowling, and Schulte-Korner [95] found them to co-occur four times as often as would be expected by chance. Dyslexia seems to be particularly associated with difficulties in memory for arithmetical facts, such as multiplication tables [96]. Indeed, Miles [97] found that 96% of a sample of eighty dyslexic 9-to-12-year-olds had significant difficulty in reciting the 6×, 7×, and 8× tables fluently.

Developmental language disorder, formerly known as specific language impairment, also often co-occurs with mathematical difficulties. Again, those aspects of mathematics that involve verbal memory are most likely to be impaired. In particular, children with developmental language disorder tend to have difficulty with verbal counting [98,99], fact retrieval [100], and transcoding, and to have much less difficulty with place value and symbolic magnitude comparison [101,102].

For example, Puvanendran, Dowker, and Demeyere [103] reported a patient with Broca’s aphasia resulting in poor verbal working memory, who demonstrated very poor fact retrieval but excellent derived fact strategy use.

Spatial abilities are usually found to be correlated with mathematical abilities [104,105], and some studies suggest that spatial training may improve children’s mathematical performance [106,107,108]. It is increasingly recognised that pattern recognition and pattern construction are significantly related to early mathematical development, and that including patterning activities in early education may facilitate mathematical learning [109,110,111,112]. There needs to be more research on individual differences in patterning, whether specific deficits occur in this area, and whether individuals with dyscalculia tend to have difficulty with patterns not involving numbers.

Attentional abilities are generally found to correlate with children’s arithmetical performance [113,114].

Most studies show that working memory is a significant predictor of arithmetic [115]. Both verbal and visuospatial working memory are generally found to be important predictors of arithmetic, though there are conflicting findings about which is a stronger predictor and how this may relate to age. Wilson and Swanson [116] found that arithmetical ability showed similar levels of correlation with working memory across a wide range of age groups, including both children and adults. At all ages, arithmetic was more closely related to verbal working memory than to visuospatial working memory. McKenzie, Bull, and Gray [117] found that visuospatial interference disrupted 6-year-olds’ arithmetic performance more than verbal interference did, whereas the reverse was true of 8-year-olds. There are, however, other studies suggesting that visuospatial working memory contributes more to older children’s arithmetic than to younger children’s arithmetic. Henry and Maclean [118] found that the strongest predictor of 11- and 12-year-olds’ arithmetical reasoning was visual memory, while this made only a small contribution to 7- and 8-year-olds’ arithmetical reasoning, which was most strongly predicted by “central executive” tasks. A meta-analysis [119] suggested a greater overall correlation between arithmetic and verbal working memory than between arithmetic and visuospatial working memory. However, this difference declined as children grew older, as correlations between arithmetic and spatial working memory remained stable over the age range, whereas correlations between arithmetic and verbal working memory declined during the primary school years.

Moreover, different aspects of working memory appear to predict different aspects of mathematical performance. Simmons, Willis, and Adams [120] gave 41 British Year 1 children (5 to 6 years old) and 49 Year 3 children (7 to 8 years old) a battery of tests of both working memory and numeracy. Working memory as a whole predicted number writing, magnitude judgment, and single-digit arithmetic. Different components of working memory were differentially important predictors of different aspects of numeracy. Visuospatial sketchpad (VSSP) functioning was an important predictor of magnitude judgments and number writing, but not a strong predictor of arithmetic. As regards specific aspects of arithmetic, central executive functioning was a significant predictor of addition accuracy in Year 1 children, while phonological loop functioning was a borderline predictor of multiplication in Year 3 children.

Allen and Dowker [121] found that visuospatial working memory predicted oral and written arithmetic in a sample of primary school children but did not predict their use of derived fact strategies. Zhang et al.’s meta-analysis [119] indicated that verbal working memory correlated more strongly with addition and subtraction than with multiplication and division.

Other executive functions appear to be strong predictors of mathematical skills. Many cross-sectional and longitudinal studies show significant relationships between executive functions and arithmetical performance [122,123,124,125,126]. Among executive functions, inhibition and shifting appear to predict arithmetic more strongly than maintenance does [5,127]. Inhibition is probably the executive function that most strongly predicts arithmetic [117,128].

The importance of inhibition is presumably because many mathematical tasks require people to selectively attend to relevant aspects of a problem and ignore irrelevant ones. This is so at all levels, not least for basic numerical tasks such as number conservation, order irrelevance, and recognising and comparing non-symbolic quantities on the basis of numerosity while ignoring numerically irrelevant perceptual cues such as area and contour. Indeed, Merkley, Thompson, and Scerif [129] found a relationship between inhibition in the animal Stroop test and numerical abilities in preschoolers. It is not clear to what extent inhibition predicts later numerical abilities by influencing early foundational abilities, versus predicting it through a direct influence on arithmetic; results are somewhat conflicting. Fuhs, Hornburg, and McNeil [130] found that that kindergarten children’s executive function predicted their arithmetic performance two years later, mainly because it predicted set size recognition, and set size estimation predicted arithmetic. On the other hand, Clayton and Gilmore [131] found that executive function predicted both quantity estimation and arithmetical skills, but quantity estimation was not significantly related to arithmetical skills. Carota et al. [30] proposed that their patient’s particular difficulty with borrowing in subtraction might be due to a deficit in the executive function of inhibition.

ADHD is usually considered to involve weaknesses in both attention and inhibition, often with associated deficits in working memory. Most studies suggest a significant comorbidity between ADHD and mathematical disabilities [132]. Attentional problems, more than hyperactivity, seem to predict mathematical difficulties.

Regardless of diagnosis, teacher ratings of attentional problems appear to specifically predict low performance in arithmetic [133,134,135]. Some studies suggest that the increased rate of mathematical difficulties in children who had been born very prematurely is due more to an increased rate of attentional difficulties than to a primary problem with numerical cognition [136]. Individuals with diagnosed ADHD tend to have more problems with those aspects of arithmetic that involve working memory (Ganor-Stern and Steinhorn, 2018 [137]), though Friedman, Rapport, Orban, et al. [138] found that, in children with ADHD, applied arithmetic problem solving was predicted by central executive abilities, rather than by phonological or visuospatial working memory.

Thus, there is a wide variety of domain-general abilities that are correlated with mathematical performance in the general population, and marked weaknesses in these abilities are often associated with mathematical difficulties. However, caution is sometimes needed in attributing mathematical difficulties to domain-general deficits, because some of the tests used to assess domain-general abilities involve some form of number processing. For example, one of the more common tests of working memory is the backward digit span test, which of course involves memory for numbers. Some studies have indeed shown that dyscalculic participants are more impaired in working memory for numbers than for words or letters [139,140]. In contrast, some other studies have shown that individuals with developmental dyscalculia have working memory deficits for other stimuli besides numbers [141,142,143].

If domain-general factors contribute to individual differences in mathematics in general and are often associated with dyscalculia, can they explain dyscalculia? In other words, is what is called dyscalculia really secondary to other deficits? The answer appears to be no. Though comorbidity is frequent, mathematical difficulties can occur independent of other deficits. Consideration of domain-general factors may enhance our understanding of dyscalculia and assist in the development of interventions to help dyscalculic individuals, but such factors cannot explain or define dyscalculia.

## 2. Discussion

It is clear that many individuals experience severe and persistent difficulties with mathematics. While some of these difficulties may result from lack of schooling, poor teaching, social disadvantage, or general learning difficulties, mathematics as such seems to present significant difficulties for many individuals.

Not all individuals with persistent mathematical difficulties can be described as dyscalculic, unless one is prepared to say that at least 20% of people are dyscalculic, given the high frequency of severe numeracy difficulties in adults [17]. There is still no definitive answer as to whether there is a distinct group that can definitely be said to be dyscalculic, what should define such a group, and whether it represents the lower end of a continuum (or perhaps any of several continua) or is qualitatively distinct.

There are two issues, in particular, that make it difficult to answer such a question: One is that arithmetical ability is not unitary but consists of multiple domain-specific components [41]. As discussed extensively in the sections above on “How does dyscalculia relate to the componential nature of arithmetical cognition?”, “How heterogeneous are deficits in dyscalculia: the importance of studying individual profiles”, and “Domain-specific factors in typical arithmetical development and in dyscalculia”, there is still significant debate about which components are most crucial in determining dyscalculia, as well as about how heterogeneous dyscalculia is, though the evidence does suggest significant heterogeneity.

The second issue is that arithmetical processing does not take place in a vacuum, separate from all other cognitive processes. As discussed in the section on “Domain-general factors in typical and atypical arithmetical development”, domain-general abilities—such as language, spatial abilities, attention, working memory, and executive functions—are significantly associated with arithmetical performance, and weaknesses in such abilities are significantly associated with difficulties in mathematics. However, mathematical difficulties sometimes occur without weaknesses in domain-general abilities: the relationship is not deterministic. There is still no consensus about whether the domain-general weaknesses, when present, contribute directly to the mathematical difficulties or are comorbid with them. Indeed, there is no consensus about the extent to which a diagnosis of dyscalculia should depend on specificity, or whether and at what point it should be ruled out if the mathematical deficits could be explained by domain-general factors.

A key issue arising from the above is the relative contribution of domain-general and domain-specific factors to mathematical development and mathematical disabilities. There is now considerable interest in the associations between mathematical performance and both domain-general and domain-specific abilities. Most studies have dealt with the extent to which children’s mathematical performance and progress are predicted by such abilities, with only relatively few such studies looking at dyscalculia or comparing children with and without mathematical disabilities. Overall, studies indicate that both domain-general and domain-specific factors are important, but it is difficult to draw firm conclusions about the relative importance of different predictors. This is because studies differ with regard to the exact domain-specific abilities being investigated, the exact domain-general abilities being investigated, the mathematical performance measures being used; the children’s age group, and the culture and school curriculum in which they are operating.

Haberstroh and Schulte-Körne [62] carried out a meta-analysis of 34 studies comparing the cognitive profiles of 8-to-12-year-old children with and without mathematical disabilities. Mathematical disabilities were defined as mathematics test scores at or below the 16th percentile, without low IQ or other comorbid disorders. In all, the analysis included 680 children with mathematical disabilities and 1565 controls. The children with mathematical difficulties performed significantly worse than the controls at calculation, arithmetic fact retrieval, number sense (including quantity processing, quantity-number linking, and numerical relations), and visuospatial short-term storage. They did not show deficits in reading or related skills, though this may reflect the fact that the study specifically excluded children with comorbid reading disabilities.

Most studies have looked at the question with regard to individual differences in the population as a whole. Chu, vanMarle, and Geary [144] carried out a longitudinal study of children’s progress in mathematics from first grade (age 6 to 7) through eighth grade (age 13 to 14). They examined the predictive role of domain-general measures (first-grade IQ and working memory scores and prior reading achievement) and domain-specific measures (prior-grade mathematics achievement and tests of prior-grade number knowledge, addition skills, and fraction knowledge). Both types of measure were important predictors. Working memory, among domain-general measures, and fraction knowledge, among domain-specific measures, became increasingly important as predictors as the children grew older. Domain-general abilities were stronger predictors than domain-specific abilities in the early grades, but the two types of predictors became equally important with increasing age.

Fuchs, Geary, Compton, et al. [145] assessed children at the beginning of first grade (approximately 6 years old) on measures of both subitising small numbers and approximate representation of large numbers, and also on measures of working memory (phonological loop, visuospatial sketchpad, and central executive), processing speed, attentive behaviour, and listening comprehension. The children were tested at the end of first grade on procedural calculations and word problems. Both domain-specific and domain-general skills were significant predictors of word problem solving, while only domain-specific skills were significant predictors of procedural calculation.

Clearly, both domain-general and domain-specific factors contribute both to dyscalculia and to individual differences in mathematical performance in the general population, though there is still a lot to be learned about the relative importance of domain-general and domain-specific factors to arithmetical performance overall, to its different components, and in different age groups.

When considering the wide variety of cognitive factors that contribute to individual differences in mathematics and to mathematical difficulties, we should note that emotional as well as cognitive factors contribute to mathematical performance. Mathematics anxiety is common and sometimes severe [146]. It is negatively associated with mathematical performance in almost all countries where it has been studied [147]. The relationship between mathematics anxiety and performance appears to be bidirectional. Despite this relationship, dyscalculia and mathematics anxiety are separate conditions. Some children with dyscalculia do not demonstrate mathematics anxiety, and some children with mathematics anxiety are highly proficient at mathematics [148]. Understanding the nature of developmental dyscalculia has important implications both for theories of the cognitive architecture of mathematical skills in typical and atypical development, and for the practice of screening and diagnosis of dyscalculia, and thereby for interventions to help individuals with dyscalculia.

From a theoretical perspective, it is important to understand to what extent dyscalculia is caused by failures of domain-specific abilities to develop; failures in domain-general cognitive processes such as long-term memory, relational integration, or verbal or spatial representations; or a failure, perhaps involving limitations in executive functions, to translate general cognitive processes into some or all domain-specific numerical abilities. We may conclude that it is likely that all of these play a role, but that primary failures in domain-specific abilities are likely to play a significant part. A greater understanding of these issues is needed both to enable a greater understanding of the independence and integration of cognitive abilities in general development and for the development of interventions for children with dyscalculia.

The topic of the development of interventions for dyscalculia and for low mathematical attainment more generally, and of whether different interventions are needed for the two, is a large topic that would need an entire article in itself. Interventions for children with mathematical difficulties have been reviewed (e.g., [149,150,151,152]). The present article refers more specifically to the implications of research on dyscalculia for the development of methods of screening and diagnosis.

While there are still many debates about the exact criteria that should be set, it is generally accepted that any screening method for dyscalculia should include multiple tests, and that no single task is sufficient. For example, Butterworth’s [153] Dyscalculia Screener tests three domain-specific abilities of number comparison, dot counting, and item-timed arithmetic. Von Aster’s [154] Neuropsychological Test Battery for Number Processing and Calculation (NUCALC/ZAREKI) includes 11 tests of domain-specific abilities: drawing 10 circles, counting dots, oral backward counting, writing numbers to dictation, reading numbers, comparing written numbers, comparing orally presented numbers, positioning numbers on an analogue scale, perceptive estimation of numerosity, contextual estimation, and word problem solving. Esmail [155] has developed a dyscalculia assessment as a part of her Dynamo Maths intervention, which includes tests of number meaning (subitising, counting, use of number symbols), number comparison (estimation, approximation, ordering, and sequencing), and number relationships (place value, number bonds and facts, mental strategies, problem solving, time measurement, and multiplication).

Beacham and Trott (2005) [156] developed a screener called DyscalculiUM, which includes a variety of abilities, all of which are specific to the domain of mathematics, but not all of which are numerical, including operational (conceptual and inferential), number comparative, number conceptual, graphical, spatiotemporal (direction and time), and symbolic abstraction.

Some screeners include both domain-specific and domain-general abilities. Zygouris, Vlachos, Dadaliaris, et al. (2017) [157] developed a screener that includes both the domain-specific abilities of calculation, numerical concepts, and problem-solving and the domain-general abilities of visual discrimination, working memory, and inhibition. Eteng-Uket (2023) [158] developed a dyscalculia test in Nigeria, which included the domain-specific abilities of number sense and arithmetic operations, and the domain-general ability of working memory.

Most diagnostic screeners today are computerised or have computerised versions (Drigas and Pappas, 2015 [159]), but recently some developers have gone beyond this in creating artificial intelligence programmes for diagnosing dyscalculia. Giri, Saini, Bhole, et al. (2020) [160] developed such a programme, which analyses the results of multiple tests (in this case, subtests of the Woodcock–Johnson Tests of Achievement, curriculum-based rests, and/or the Wide Range Achievement Test), assigns weights to the different scores, and uses them to calculate the probability that an individual is dyscalculic. Such programmes may reduce the time, labour, and possibilities for error that could result from the manual analysis of a combination of multiple tests.

## 3. Conclusions

There is still much controversy about the nature and distinctness of developmental dyscalculia, though there is much evidence that many children and adults experience marked difficulties in arithmetic. However, the study of both acquired and developmental dyscalculia, as well as of individual differences in mathematical ability in the general population, indicates that arithmetic is made up of numerous components, and that people cannot simply be described as “good” or “bad” at arithmetic. This has important implications both for the development of targeted interventions for children struggling with mathematics, and for general educational practice.

There is still significant debate about which components are most likely to be impaired in developmental dyscalculia, but most studies show impairment in symbolic number processing, and many show impairment in subitising, in approximate non-symbolic number processing, or both. Studies also show that individuals with dyscalculia also often show deficits in domain-general abilities, such as spatial abilities, working memory, and executive functions such as inhibition. However, it is unclear whether such deficits contribute directly to dyscalculia or are comorbid with it.

It is also unclear whether dyscalculia is qualitatively distinct from the general continuum of mathematical ability or represents the lowest part of that continuum. However, it is clear that there are many parallels among the associations and discrepancies between different components of mathematical ability that are often found in typically achieving individuals and those that are found in dyscalculia. In order to gain a greater understanding both of individual differences in the general population and of dyscalculia, we must integrate our research on both topics. We cannot fully understand “typical” individual differences without understanding dyscalculia, and we cannot fully understand dyscalculia without understanding individual differences.

## 4. Further Directions

As can be seen, there is a great deal more to be learned about the nature and causes of developmental dyscalculia and the extent to which it relates to the typical range of individual differences in arithmetic. We still do not know whether developmental dyscalculia represents the lower end of the continuum of mathematical abilities—and, if so, where the cut-off point should be set—or whether at least some individuals have a specific and separate condition that does not fit on the usual continuum at all. Given the componential nature of numerical cognition, we need to know more about the extent to which dyscalculia mainly stems from deficits in particular components and, if so, which ones. In view of the frequent comorbidity of dyscalculia with other specific learning disabilities, along with its frequent association with other deficits, we need to know more about whether dyscalculia that is completely specific differs in nature from dyscalculia that is comorbid with other deficits.

We also need to know more about whether developmental dyscalculia is more influenced by domain-specific or domain-general abilities, which particular domain-specific and/or domain-general abilities are most important, and to what extent domain-specific and domain-general abilities may interact in causing (and influencing the course of) dyscalculia. It is important to investigate whether any such influences and interactions reflect associations between mathematical performance and particular domain-specific and domain-general abilities throughout the range of mathematical ability/abilities, or whether they are relatively specific to dyscalculia.

At present, rather little is known about how developmental dyscalculia relates to characteristics of brain structure and function, and particularly whether it is associated with reduced or excessive connectivity between brain areas involved in mathematical cognition. Much more work needs to be carried out on this topic, and on whether any such brain characteristics vary with individual differences in mathematics, or any of its components, in the general population.

Crucially, we need to know whether and to what extent answers to these theoretically important questions are also of practical and educational importance. To what extent do specificity, severity, and relation to the overall continuum of mathematical ability predict dyscalculic individuals’ likely response to intervention in general, or the particular type of intervention that is most likely to be effective?

Finally, it should be pointed out that almost all research has been on deficits in numerical ability. Indeed, following common but perhaps misleading practice, this chapter has used the terms “mathematics” and “arithmetic” virtually interchangeably. To what extent may we find deficits in other areas of mathematics, such as geometry, measurement, and algebra, and to what extent do these co-occur with numerical and arithmetical disabilities? This is clearly an important topic for future research.
